# Potential determinants of vitamin D in Finnish adults: a cross-sectional study from the Northern Finland birth cohort 1966

**DOI:** 10.1136/bmjopen-2016-013161

**Published:** 2017-03-06

**Authors:** Saranya Palaniswamy, Elina Hyppönen, Dylan M Williams, Jari Jokelainen, Estelle Lowry, Sirkka Keinänen-Kiukaanniemi, Karl-Heinz Herzig, Marjo-Riitta Järvelin, Sylvain Sebert

**Affiliations:** 1 Biocenter Oulu, University of Oulu, Oulu, Finland; 2 Faculty of Medicine, Center for Life Course Health Research, University of Oulu, Oulu, Finland; 3 Centre for Population Health Research, School of Health Sciences and Sansom Institute, University of South Australia, South Australian Health and Medical Research Institute, Adelaide, Australia; 4 Population, Policy and Practice, Institute of Child Health, University College London, London, UK; 5 Department of Medical Epidemiology and Biostatistics, Karolinska Institutet, Stockholm, Sweden; 6 MRC and Unit of Primary Care, Oulu University Hospital, Oulu, Finland; 7 Department of Physiology, Institute of Biomedicine, University of Oulu, Oulu, Finland; 8 Department of Gastroenterology and Metabolism, Poznan University of Medical Sciences, Poznan, Poland; 9 CEU Cardenal Herrera University, Valencia, Spain; 10 Department of Epidemiology and Biostatistics, School of Public health, Imperial College London, London, UK; 11 MRC-PHE Centre for Environment and Health, School of Public Health, Imperial College London, London, UK; 12 Department of Genomics of Complex Diseases, School of Public Health, Imperial College London, London, UK

**Keywords:** Vitamin D, 25-hydroxyvitamin D, Northern Finland

## Abstract

**Objective:**

Evidence from randomised controlled trials suggests that vitamin D may reduce multimorbidity, but very few studies have investigated specific determinants of vitamin D2 and D3 (two isoforms of 25-hydroxyvitamin D). The aim of the study was to investigate the determinants of vitamin D2 and D3 and to identify the risk factors associated with hypovitaminosis D.

**Design:**

Cross-sectional study.

**Setting:**

Northern Finland Birth Cohort 1966.

**Participants:**

2374 male and 2384 female participants with data on serum 25(OH)D_2_ and 25(OH)D_3_ concentrations measured at 31 years of age (1997), together with comprehensive measures of daylight, anthropometric, social, lifestyle and contraceptive cofactors.

**Methods:**

We assessed a wide range of potential determinants prior to a nationwide fortification programme introduced in Finland. The determinants of 25(OH)D_2_, 25(OH)D_3_ and 25(OH)D concentrations were analysed by linear regression and risk factors for being in lower tertile of 25(OH)D concentration by ordinal logistic regression.

**Results:**

At the time of sampling, 72% of the participants were vitamin D sufficient (≥50 nmol/L). Low sunlight exposure period (vs high) was associated positively with 25(OH)D_2_ and negatively with 25(OH)D_3_ concentrations. Use of oral contraceptives (vs non-users) was associated with an increase of 0.17 nmol/L (95% CI 0.08 to 0.27) and 0.48 nmol/L (95% CI 0.41 to 0.56) in 25(OH)D_2_ and 25(OH)D_3_ concentrations. Sex, season, latitude, alcohol consumption and physical activity were the factors most strongly associated with 25(OH)D concentration. Risk factors for low vitamin D status were low sunlight exposure defined by time of sampling, residing in northern latitudes, obesity, higher waist circumference, low physical activity and unhealthy diet.

**Conclusions:**

We demonstrate some differential associations of environmental and lifestyle factors with 25(OH)D_2_ and 25(OH)D_3_ raising important questions related to personalised healthcare. Future strategies could implement lifestyle modification and supplementation to improve vitamin D2 and D3 status, accounting for seasonal, lifestyle, metabolic and endocrine status.

**Strengths and limitations of this study**

- Data were from a large homogeneous Northern Finnish Birth Cohort (latitude ≥65°N) and included information on several determinants of 25(OH)D_2_ and 25(OH)D_3_ in young adults.
- The sample was collected in Finland before the implementation of national policy on fortification of milk and margarine with vitamin D.
- This is the first study to report the influence of oral contraceptive pills on 25(OH)D_2_ and 25(OH)D_3_ concentrations.
- The finding offers an independent replication of the differential associations of seasonality with serum 25(OH)D_2_ and 25(OH)D_3_ concentrations, as previously observed in British children supporting evidence for different biological pathways regulating vitamin D2 and D3 status.
- Limitations of the study include lack of a more precise measure of UV-B exposure, information on whether study participants were taking vitamin D supplementation, detailed dietary index and information on outdoor and indoor physical activity which could help account for residual confounders.

## Introduction

Serum 25-hydroxyvitamin D (25(OH)D), the circulating biomarker of vitamin D status, is found to be associated with multiple pathological conditions.^1–4^ There is growing interest in understanding the causal role of vitamin D in the aetiology of chronic metabolic diseases including obesity,^1^ ^2^ type 2 diabetes^3^ and mortality.^4^ Vitamin D is classified as a pro-hormone which exists in circulation in two major forms of 25(OH)D: 25(OH)D_2_ (ergocalciferol) and 25(OH)D_3_ (also known as cholecalciferol).^5^ ^6^ Serum 25(OH)D_2_ is obtained only from plant-derived dietary sources, fortification or supplementation.^5^ ^7^ In contrast, 25(OH)D_3_ is predominantly obtained from sunlight exposure and smaller quantities from dietary sources such as fatty fish, fortified milk products and supplements.^5^ ^6^ In Finland, the milk products and spreadable fats are fortified with 25(OH)D_3_.^8^ The current fortification contains 25(OH)D_3_ due to somewhat lower biopotency of 25(OH)D_2_ that requests further understanding.^8^ Vitamin D status is determined by measuring 25(OH)D,^7^ which reflects the combined intake of vitamins 25(OH)D_2_ and 25(OH)D_3_ and subcutaneous synthesis during the past 3–4 weeks.^5^ ^9^ ^10^

There is limited knowledge about the factors associated with each isoform that may have differential environmental determinants.^10^ Total 25(OH)D and the relative proportions of 25(OH)D_2_ and 25(OH)D_3_ are suggested to reflect a number of health and lifestyle factors that might be sex specific.^11^ ^12^ In young adults, lifestyle and body composition differ between men and women.^12^ ^13^ As to whether the differential composition of the body between sexes, as well as other endocrine factors, will be reflected by differences in the 25(OH)D concentration and the 25(OH)D_2_ and 25(OH)D_3_ components is yet unknown.^12^ ^13^ There are no previous comprehensive studies examining the factors associated with 25(OH)D_2_ and 25(OH)D_3_ concentrations in Finland. This limits the availability of inferences that could help to identify people at risk of vitamin D deficiency, and improved fortification policies to meet the requirements of those living at northern latitudes.^8^ ^14^

We examined here factors associated with 25(OH)D_2_, 25(OH)D_3_ and total 25(OH)D concentrations in Finnish adults aged 31 years prior to the implementation of a nationwide supplementation of vitamin D via fortification of milk products and margarine in 2002.^8^ ^14^

## Methods

### Study population

We analysed data on participants from the Northern Finland Birth Cohort 1966 (NFBC1966) which has previously been described in detail.^15^ ^16^ In brief, all women who were pregnant, residing in Northern Finland (provinces of Oulu and Lapland) with expected dates of delivery between 1 January and 31 December,1966 were targeted for enrolment in the study. Over 96% of eligible women participated. This comprised of 12 055 mothers and 12 058 live born children. The children were followed up at regular intervals from birth onwards. In 1997, when participants were aged 31 years, all cohort participants with known addresses in the provinces of Oulu and Lapland (65°N to 70°N) and in Helsinki (60°N) area were sent a postal questionnaire and invited to a clinical examination which also included, a fasted blood sample.^17^ A total of N=4758 individuals of white European origin were included in the study as shown in online supplementary figure S1. All participants gave written informed consent. The procedures follow the 1964 Helsinki declaration and its later amendments or comparable ethical standards. The present study includes individuals with a complete set of data on variables of interest, as detailed below.

10.1136/bmjopen-2016-013161.supp1 supplementary figure

### Outcome variables

#### 25(OH)D measurement

Serum 25(OH)D_2_ and 25(OH)D_3_ were measured by liquid chromatography tandem mass spectrometry and the detailed assay procedure is published elsewhere.^18^ Participants with 25(OH)D_2_ values under the detectable limit were assigned a value of 1.25 nmol/L.^18^ Total 25(OH)D is obtained as the actual sum of D2+D3 without 25(OH)D_2_ low value assignment. Consequently, in the tables, total 25(OH)D may differ slightly from exact sum of D2 and D3. Vitamin D sufficiency criteria were defined according to the Institute of Medicine (IOM) guidelines as ≤30 nmol/L (risk/deficiency), 30–50 nmol/L (risk/insufficiency) and ≥50 nmol/L (sufficient).^19^

### Explanatory factors

The season of participant attendance at the clinical assessment was categorised according to the Finnish Meteorological Institute standard as high sunlight (summer (1 June–30 August) autumn (1 September–31 October)) and low sunlight season (winter (1 November–31 March) and spring (1 April–31 May)).^20^ This definition aims to assess the impact of natural high and low vitamin D level periods throughout the calendar year. The residence of the participants at age 31 years was collected from the population register office. They were categorised as residing in Helsinki (60°N); the city of Oulu (65°N) and elsewhere in northernmost provinces of Oulu and Lapland (>65°N). In Helsinki, blood samples were collected only during winter in contrast to all year round in other provinces, due to the feasibility of data collection and were excluded in multivariable analyses. Height (cm) and weight (kg) were measured in barefoot and loose clothing by well-trained nurses. Body Mass Index (BMI) (kg/m^2^) was calculated and categorised according to the WHO 1998.^21^ Waist circumference (cm) was categorised as elevated when it was ≥94 cm in men and ≥80 cm in women.^22^

Categorisation of following lifestyle variables was based on the responses in the postal questionnaire. Current smoking was categorised as non-smoker, former/occasional or active smoker. Alcohol consumption during the 6 months prior to the questionnaire was calculated as grams per day (g/day) and has been described elsewhere.^23^ It was further categorised according to WHO sex-specific classification as abstainer, low-risk drinker (≤20 and ≤40 g/day for women and men, respectively) or at-risk drinker (>20 and >40 g/day for women and men, respectively).^24^ The frequency of computer use during leisure time was categorised as never, no more than once per week, on 2–5 days per week or on more than 5 days per week. The reported frequency and duration of leisure time and brisk physical activity were used to calculate the metabolic equivalent of task (MET) scores in hours per week, and these were ordered into quartiles. An intensity value of 3 METs is considered as light physical activity, and 5 METs as brisk physical activity.^25^ Diet score was calculated based on the consumption of various food in the previous 6 months and was reported on a structured six-point scale (from never/<once per month to several times per day) and has been described previously.^23^ The food frequency question included 32 products categorised under grain products, milk products, vegetables, meat, fruits and others (chocolates, sweets and packaged meals). An unhealthy diet included daily or frequent consumption of red meat and less frequent consumption of rye or crisp bread, berries or fruit, salads and vegetables. The score ranged from 0–5 and was categorised as healthy diet (<3 points) and unhealthy diet (4–5 points).^23^ Current use of contraception by women was categorised as no contraception use, other methods of contraception (hormone intrauterine device (IUD), copper IUD, chemical contraception) or oral contraceptive pill (OCP).^26^ Socioeconomic position (SEP) was categorised as I and II (professional), III (skilled worker), IV (unskilled worker), V (farmer) and VI (others-pensioner, student, long-term unemployed or not defined). The exclusion criteria consisted of participants with non-fasting blood samples, pregnant women, no consent for use of data and persons whose information was missing on one or more variables of interest.

### Statistical analyses

All statistical analyses were performed using SAS V.9.4 (SAS Institute, Cary, NC, USA). The variables were assessed for normality and log transformed where relevant. Mean differences between sexes for continuous variables were measured by independent samples t-test and analysis of variance; and Pearson χ^2^ test for categorical variables. We performed univariable linear regression analysis to explore the association between explanatory variables and serum 25(OH)D_2_, 25(OH)D_3_ and total 25(OH)D concentrations. We log transformed 25(OH)D_2_, 25(OH)D_3_ and 25(OH)D, and expressed these on standardised scales (z-scores). To examine whether sex was an effect modifier of associations, an interaction term (sex × explanatory variable) was additionally included in univariable analyses. We conducted multivariable analyses aiming to examine mutually adjusted associations of different exposures with 25(OH)D_2_, 25(OH)D_3_ and 25(OH)D measures, namely season of blood sampling (low and high sunlight period), latitude, BMI, waist circumference, SEP, smoking status, alcohol consumption, leisure time computer use, physical activity, diet score and contraception status. In addition, we examined serum 25(OH)D_2_, 25(OH)D_3_ and 25(OH)D concentrations by excluding women using OCPs.

Following examination of the determinants associated with 25(OH)D_2_, 25(OH)D_3_ and 25(OH)D concentrations, we performed multinomial ordinal logistic regression analysis to assess the risk factors associated with being in the lower tertile (reference: tertile III) of vitamin D. Owing to equivocal definitions of cut-off values for vitamin D status in the general population, we categorised the analysis sample into tertiles of 25(OH)D. Statistical significance was set at global p<0.05 using two-tailed test.

## Results

The characteristics of the study population at age 31 years are summarised in table 1. According to Institute of Medicine (IOM) criteria for vitamin D sufficiency, 3.3% were deficient, 24.2% were insufficient and 71.5% were sufficient. A total of 3.0% of men and 3.5% of women were deficient. Serum D2 concentrations were lower in men when compared with women. However, the mean serum D3 and total 25(OH)D concentrations tended to be higher in men than in women, although the difference was not statistically significant. Though, the difference became more pronounced after excluding women using OCPs. There were no interactions observed by sex with any explanatory variables (p for interactions >0.05, data not shown).

**Table 1:** Characteristics of the study population*

	Total		Male		Female		p Value
Sample size (n)	4758		2374		2384		
	n or mean	% or 95% CI	n or mean	% or 95% CI	n or mean	% or 95% CI	
Daylight							
Season of blood sampling† (n %)							
High sunlight	2953	62.1	1501	63.2	1452	60.9	0.09
Low sunlight	1805	37.9	873	36.8	932	39.1	
Latitude‡ (n %)							
65°N	891	28.7	460	29.3	431	28.1	0.58
>65°N	3105	71.3	1571	70.7	1534	71.9	
Anthropometry							
BMI (kg/m^2^) (mean, 95% CI)	24.7	24.6 to 24.8	25.2	25.1 to 25.3	24.1	23.9 to 24.3	<0.01
Waist circumference(cm) (mean, 95% CI)	83.8	83.5 to 84.2	88.9	88.5 to 89.3	78.8	78.3 to 79.2	<0.01
Socioeconomic position: (n %)							
I+II (Professional)	1134	23.8	653	27.5	481	20.2	<0.01
III (Skilled worker)	1483	31.2	433	18.2	1050	44.0	
IV (Unskilled worker)	1228	25.8	856	36.1	372	15.6	
V (Farmer)	165	3.5	111	4.7	54	2.3	
VI (Other)	748	15.7	321	13.5	427	17.9	
Lifestyle							
Smoking (n %)							
Non-smoker	2128	44.7	952	40.1	1176	49.4	<0.01
Former/occasional smoker	1214	25.5	600	25.3	614	25.7	
Active smoker	1416	29.8	822	34.6	594	24.9	
Alcohol consumption (g/day) (n %)							
Abstainer	426	8.95	191	8.1	235	9.9	<0.01
Low-risk drinker	4053	85.18	2026	85.3	2027	85.0	
At-risk drinker	279	5.86	157	6.6	122	5.1	
Leisure time computer use (n %)							
Never	1708	35.9	852	35.9	856	35.9	<0.01
No more than once per week	691	14.5	312	13.1	379	15.9	
On 2 to 5 days per week	1419	29.8	656	27.6	763	32.0	
On more than 5 days per week	940	19.8	554	23.4	386	16.2	
Physical activity (MET hours/week) (mean, 95% CI)	15.0	14.6 to 15.4	14.9	14.4 to 15.6	15.0	14.5 to 15.6	<0.01
Diet score (n %)							
0–1	1461	30.71	453	19.1	1008	42.3	<0.01
2–3	2739	57.57	1531	64.5	1208	50.6	
4–5	558	11.73	390	16.4	168	7.1	
Contraception status§ (n %)							
No contraception					1154	49.1	
Other kinds of contraception					591	25.1	
Oral contraceptive pills (OCP)					607	25.8	
Vitamin D status (mean, 95% CI)							
Serum total 25(OH)D¶	68.4	67.6 to 69.2	68.9	67.7 to 70.1	67.9	66.7 to 68.9	0.78
Serum 25(OH)D_3_	64.8	63.9 to 65.6	65.6	64.4 to 66.7	64.0	62.8 to 65.1	0.45
Serum 25(OH)D_2_	4.2	3.9 to 4.3	3.9	3.6 to 4.2	4.4	4.1 to 4.7	<0.01
Vitamin D status without OCP** (mean, 95% CI)							
Serum total 25(OH)D¶	67.0	66.2 to 67.9	68.9	67.7 to 70.1	64.6	63.3 to 65.8	<0.01
Serum 25(OH)D_3_	63.6	62.8 to 64.5	65.6	64.4 to 66.7	60.9	59.8 to 62.2	<0.01
Serum 25(OH)D_2_	4.0	3.8 to 4.2	3.9	3.6 to 4.2	4.2	3.9 to 4.5	0.05

Values are presented as mean, 95% CIs or number (%).

*p Value was calculated using independent samples *t-*test for normally distributed variables and Pearson's χ^2^ test for categorical variables.

†The season of blood sampling were categorised as high sunlight (summer (1 June–30 August), autumn (1 September–31 October)) and low sunlight (winter (1 November–31 March) and spring (1 April–31 May)).

‡Data included only on samples taken during all seasons from Oulu city and other provinces of Oulu and Lapland. Data not included on N=343 in men and N=419 in women with samples taken during winter months from Helsinki region.

§Data available on N=2352 individuals (N=32 missing with contraception status in women).

¶Serum total 25(OH)D may differ slightly from the actual sum of D2 and D3 because of amendment of undetectable D2 values (see methods).

**Data on N=607 using oral contraceptives excluded.

BMI, body mass index; MET, metabolic equivalent of task of physical activity; 25(OH)D, 25-hydroxyvitamin D; 25(OH)D_2_, ergocalciferol; 25(OH)D_3_, cholecalciferol.

### Risk factors associated with lower vitamin D status according to tertile distribution

**Figure 1:**
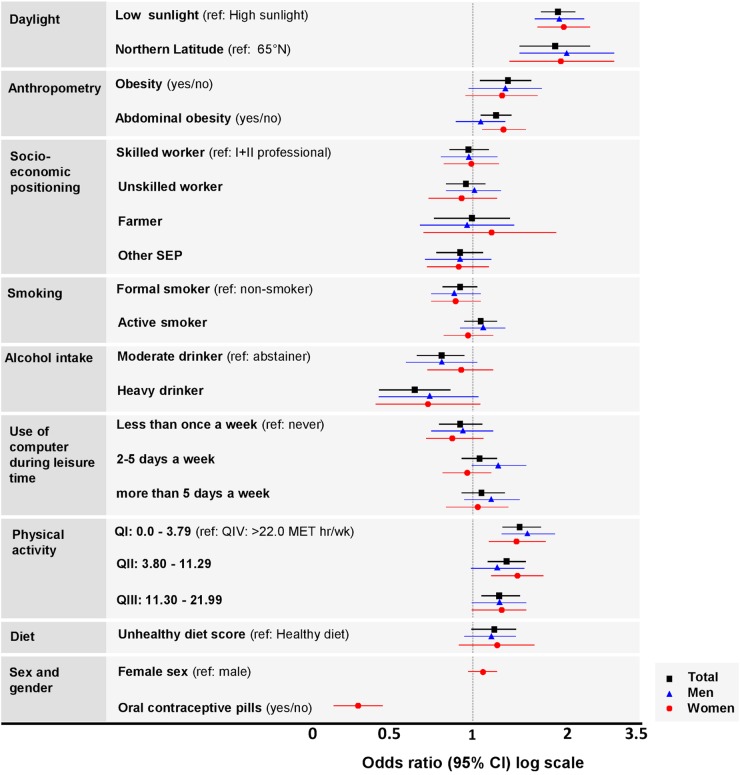
Forest plots showing the risk factors associated with low vitamin D status based on tertile distribution in the total population and by sex. Associations from mutually adjusted ordinal logistic regression ORs (on log scale) show the risk of being in the lower vitamin D tertile.

Characteristics of the study population across the tertiles of serum 25(OH)D concentration are summarised in table 2 (total), online supplementary tables S1 and S2 (men and women, respectively). Unadjusted and adjusted ordinal logistic regression analyses for the odds of being in the lower tertiles of 25(OH)D compared with the highest are shown in online supplementary table S3. The mutually adjusted model shows the risk of being in lower tertile of 25(OH)D was increased in individuals whose blood samples were collected during low sunlight months, living in higher latitudes, having elevated waist circumference and unhealthy diet. Figure 1 illustrates the mutually adjusted analyses with OR estimates for the impact of daylight, anthropometric, social and lifestyle risk factors for being in vitamin D tertile I (low) compared with tertile III (high). In sex-stratified analysis, women using OCPs had reduced odds of being in the tertile I (low) of 25(OH)D. The mean vitamin D concentration was ∼10% higher in OCP users (vs non-users).

**Table 2:** The characteristic of all Northern Finland Birth Cohort (NFBC) 1966 participants (N=4758) in the present study at 31 years by serum 25-hydroxyvitamin D tertiles* (I= the lowest tertile; III= the highest tertile)

	I		II		III		
Tertile of serum 25(OH)D† N	1592		1589		1577		
	n or mean	% or 95% CI	n or mean	% or 95% CI	n or mean	% or 95% CI	p Value
Sex n %							
Males	782	32.9	800	33.7	792	33.4	0.75
Females	810	33.9	789	33.2	785	32.9	
Environmental factors							
Season of blood drawn‡ n %							
High sunlight	566	19.2	1012	34.3	1375	46.5	<0.0001
Low sunlight	1026	56.9	577	31.9	202	11.2	
Latitude§ n %							
65°N	210	23.6	305	34.2	376	42.2	0.0006
>65°N	923	29.7	1042	33.6	1140	36.7	
Anthropometry							
Body mass index (kg/m^2^) mean 95% CI	24.8	24.6 to 25.0	24.8	24.6 to 24.9	24.4	24.2 to 24.6	0.017
Waist circumference (cm) mean 95% CI	84.6	83.9 to 85.2	84.0	83.4 to 84.6	82.9	82.3 to 83.4	0.0003
Socioeconomic position n %							
I+II (Professional)	421	37.2	374	32.9	339	29.9	0.0046
III (Skilled worker)	501	33.8	503	33.9	479	32.3	
IV (Unskilled worker)	386	31.4	427	34.8	415	33.8	
V (Farmer)	60	36.4	49	29.7	56	33.9	
VI (Other)	224	29.9	236	31.6	288	38.5	
Lifestyle factors							
Smoking n %							
Non-smoker	742	34.9	686	32.2	700	32.9	0.055
Former/occasional smoker	366	30.2	438	36.1	410	33.7	
Active smoker	484	34.2	465	32.9	467	32.9	
Alcohol consumption (g/day) n %							
Abstainer	165	38.7	146	34.3	115	27.0	0.053
Low risk drinker	1335	32.9	1349	33.3	1369	33.8	
At-risk drinker	92	32.9	94	33.7	93	33.4	
Leisure time computer use n %							
Never	537	31.4	599	35.1	572	33.5	0.0012
No more than once per week	208	30.1	234	33.9	249	36.0	
On 2 to 5 days per week	487	34.3	447	31.5	485	34.2	
On more than 5 days per week	360	38.3	309	32.9	271	28.8	
Quartile of physical activity (MET hours per week) n %							
QI: 0.0–3.79	444	36.6	394	32.5	376	30.9	<0.0001
QII: 3.80–11.29	403	33.9	421	35.4	365	30.7	
QIII: 11.30–21.99	415	34.5	397	33.0	391	32.5	
QIV: >22.0	330	28.7	377	32.7	445	38.6	
Diet score n %							
0–1	478	32.7	477	32.7	506	34.6	0.26
2–3	912	33.3	920	33.6	907	33.1	
4–5	202	36.2	192	34.4	164	29.4	
Females only							
Contraception n %							
No contraception	441	38.2	401	34.8	312	27.0	<0.001
Other kinds of contraception	216	36.6	187	31.6	188	31.8	
Oral contraceptive pills	140	23.1	190	31.3	277	45.6	

The values are expressed as mean and 95% CIs; numbers and %.

*Differences between males and females were tested with ANOVA for normally distributed variables and Pearson's χ^2^ test for categorical variables.

†Mean (95% CI) of 25-hydroxyvitamin D tertiles for all were 41.50 (41.11 to 41.89), 63.87 (63.55 to 64.19) and 100.01 (98.81 to 101.22). Serum total 25(OH)D may differ slightly from the actual sum of D2 and D3 because of amendment of undetectable D2 values (see methods).

‡The season of blood sampling were categorised as high sunlight (summer (1 June–30 August), autumn (1 September–31 October)) and low sunlight (winter (1 November–31 March) and spring (1 April–31 May)).

§Data included only on samples taken during all seasons from Oulu city and other provinces of Oulu and Lapland. Data not included on N=343 in men and N=419 in women with samples taken during winter months from Helsinki region.

MET, metabolic equivalent of task of physical activity; 25(OH)D, 25-hydroxyvitamin D;

10.1136/bmjopen-2016-013161.supp2 supplementary tables

### Factors associated with serum 25(OH)D_2_, 25(OH)D_3_ and 25(OH)D concentrations

Univariable and multivariable associations of daylight, anthropometric, social and lifestyle factors with 25(OH)D_2_, 25(OH)D_3_ and 25(OH)D in the total population are shown in table 3, online supplementary tables S4 and S5 (men and women, respectively). The factors associated with 25(OH)D_2_ and 25(OH)D_3_ were sex, season of blood sampling, latitude, obesity, waist circumference and physical activity. Unhealthy diet and active smoking were univariably associated with lower 25(OH)D_2_ concentrations; and SEP was associated univariably with lower 25(OH)D_3_ concentrations.

**Table 3:** Major factors associated with serum 25(OH)D_2_ (vitamin D2), 25(OH)D_3_ (vitamin D3) and total 25(OH)D (vitamin D) nmol/L concentrations assessed by univariable and multiple linear regression analysis, total (N=4758)*

Explanatory variables	Serum 25(OH)D_2_, nmol/L†				Serum 25(OH)D_3_, nmol/L†				Serum 25(OH)D, nmol/L†			
	Univariable		Multivariable‡		Univariable		Multivariable‡		Univariable		Multivariable‡	
	Β	95% CI	β	95% CI	β	95% CI	β	95% CI	β	95% CI	β	95% CI
Sex (reference: males)												
Females	0.10	0.04 to 0.16	0.12	0.06 to 0.18	−0.06	−0.12 to −0.003	−0.09	−0.14 to −0.04	−0.04	−0.09 to 0.02	−0.06	−0.12 to −0.01
Global p value		0.0008		0.0001		0.038		0.0005		0.21		0.019
Daylight												
Season of blood sampling § (reference: high sunlight)												
Low sunlight	0.57	0.51 to 0.63	0.29	0.21 to 0.36	−1.03	−1.08 to −0.98	−0.43	−0.49 to −0.36	−0.92	−0.97 to −0.87	−0.36	−0.42 to −0.29
Global p value		<0.0001		<0.0001		<0.0001		<0.0001		<0.0001		<0.0001
Latitude (reference: 65°N)												
>65°N	−0.08	−0.16 to −0.01	−0.06	−0.13 to 0.02	−0.14	−0.21 to −0.07	−0.18	−0.24 to −0.12	−0.16	−0.23 to −0.08	−0.20	−0.26 to −0.13
Global p value		0.023		0.12		0.0002		<0.0001		<0.0001		<0.0001
Anthropometry												
BMI (kg/m^2^) (reference : normal (18.5–24.99))												
Underweight (<18.5)	−0.05	−0.25 to 0.15	−0.06	−0.24 to 0.13	−0.08	−0.27 to 0.12	−0.06	−0.22 to 0.10	−0.09	−0.29 to 0.11	−0.08	−0.25 to 0.09
Overweight (25–29.99)	−0.10	−0.17 to −0.04	−0.01	−0.08 to 0.06	0.02	−0.04 to 0.08	−0.001	−0.06 to 0.06	−0.004	−0.07 to 0.06	−0.005	−0.07 to 0.06
Obese (≥30)	−0.13	−0.24 to −0.03	−0.01	−0.14 to 0.11	−0.19	−0.30 to −0.09	−0.16	−0.27 to −0.06	−0.23	−0.33 to −0.12	−0.17	−0.27 to −0.06
Global p value		0.0035		0.94		0.0008		0.0035		0.0002		0.0057
Waist circumference (cm) (reference: m<94, f<80)												
M≥94, F≥80	−0.09	−0.15 to −0.03	−0.10	−0.18 to −0.02	−0.13	−0.19 to −0.07	−0.05	−0.12 to 0.01	−0.15	−0.21 to −0.09	−0.08	−0.15 to −0.01
Global p value		0.003		0.013		<0.0001		0.11		<0.0001		0.030
Socioeconomic position (reference: I+II (professional))												
III (Skilled worker)	−0.05	−0.13 to 0.03	−0.05	−0.13 to 0.02	0.08	0.001 to 0.15	0.03	−0.04 to 0.09	0.07	−0.003 to 0.15	0.03	−0.04 to 0.09
IV (Unskilled worker)	−0.06	−0.15 to 0.02	0.01	−0.07 to 0.10	0.14	0.06 to 0.22	0.02	−0.05 to 0.09	0.12	0.04 to 0.21	0.03	−0.05 to 0.10
V(Farmer)	−0.11	−0.27 to 0.06	−0.02	−0.18 to 0.14	0.06	−0.10 to 0.22	−0.06	−0.19 to 0.08	0.03	−0.13 to 0.20	−0.06	−0.20 to 0.08
VI(Other)	−0.14	−0.23 to −0.05	−0.06	−0.16 to 0.03	0.21	0.11 to 0.29	0.05	−0.03 to 0.13	0.18	0.09 to 0.28	0.05	−0.03 to 0.13
Global p value		0.056		0.33		0.0002		0.49		0.0012		0.56
Lifestyle												
Smoking (reference: non-smoker)												
Former/occasional smoker	−0.03	−0.10 to 0.04	−0.01	−0.08 to 0.06	0.05	−0.02 to 0.12	0.02	−0.03 to 0.08	0.04	−0.03 to 0.11	0.02	−0.04 to 0.08
Active smoker	−0.10	−0.17 to −0.03	−0.05	−0.12 to 0.02	0.007	−0.06 to 0.07	−0.05	−0.10 to 0.01	−0.02	−0.08 to 0.05	−0.06	−0.12 to 0.0002
Global p value		0.014		0.37		0.39		0.071		0.37		0.051
Alcohol consumption (g/day) (reference: abstainer)												
Low risk drinker	0.04	−0.06 to 0.14	0.07	−0.03 to 0.16	0.17	0.07 to 0.27	0.12	0.04 to 0.20	0.19	0.09 to 0.29	0.14	0.06 to 0.23
At-risk drinker	0.03	−0.12 to 0.18	0.07	−0.08 to 0.21	0.13	−0.02 to 0.28	0.19	0.06 to 0.31	0.14	−0.02 to 0.29	0.20	0.07 to 0.33
Global p value		0.71		0.39		0.0043		0.0041		0.0012		0.0019
Leisure time computer use (reference : never)												
No more than once per week	0.03	−0.06 to 0.12	0.002	−0.08 to 0.09	0.01	−0.08 to 0.09	0.02	−0.05 to 0.09	0.01	−0.08 to 0.10	0.02	−0.06 to 0.09
On 2 to 5 days per week	0.03	−0.04 to 0.10	−0.01	−0.08 to 0.06	−0.04	−0.11 to 0.03	−0.03	−0.09 to 0.03	−0.03	−0.10 to 0.04	−0.03	−0.09 to 0.03
On more than 5 days per week	0.09	0.01 to 0.17	0.02	−0.07 to 0.10	−0.20	−0.28 to −0.12	−0.09	−0.16 to −0.02	−0.17	−0.25 to −0.10	−0.08	−0.15 to −0.01
Global p value		0.14		0.93		<0.0001		0.026		<0.0001		0.10
Quartile of physical activity (MET-hours per week) (reference: QI: 0.0–3.79)												
QII: 3.80–11.29	0.08	0.0003 to 0.16	0.05	−0.03 to 0.12	−0.02	−0.10 to 0.06	0.003	−0.06 to 0.07	−0.01	−0.09 to 0.07	0.01	−0.06 to 0.07
QIII: 11.30–21.99	0.10	0.02 to 0.18	0.05	−0.03 to 0.12	0.02	−0.06 to 0.10	0.05	−0.01 to 0.12	0.04	−0.04 to 0.12	0.07	−0.002 to 0.13
QIV: >22.0	0.11	0.03 to 0.20	0.08	−0.002 to 0.16	0.15	0.07 to 0.23	0.14	0.07 to 0.20	0.18	0.10 to 0.26	0.16	0.09 to 0.23
Global p value		0.022		0.29		<0.0001		<0.0001		<0.0001		<0.0001
Diet score (reference: healthy diet)												
Unhealthy diet	−0.12	−0.21 to −0.03	−0.06	−0.15 to 0.02	−0.07	−0.15 to 0.02	−0.07	−0.15 to −0.0004	−0.10	−0.18 to −0.01	−0.09	−0.17 to −0.01
Global p value		0.009		0.14		0.15		0.049		0.034		0.022

*The values are standardised regression coefficients (β) and p values from linear regression models by entering each variable separately in univariable analysis and by entering all the variables in multivariable analysis.

†1 SD increase/decrease in 25(OH)D_2_, 25(OH)D_3_ and 25(OH)D nmol/L per 1 unit or category change in explanatory variable.

‡Analysis performed on N=3996 (total). Blood drawn only in winter on N=343 men and N=419 in women residing in Helsinki were excluded.

§The season of blood sampling were categorised as high sunlight (summer (1 June–30 August), autumn (1 September–31 October)) and low sunlight (winter (1 November–31 March) and spring (1 April–31 May)).

MET, metabolic equivalent of task of physical activity; 25(OH)D, 25-hydroxyvitamin D; 25(OH)D_2_, ergocalciferol; 25(OH)D_3,_ cholecalciferol.

In multivariable analyses, sex was associated with serum 25(OH)D_2_ and 25(OH)D_3_ concentrations. Men had 0.5 nmol/L lower 25(OH)D_2_ but 1.6 nmol/L higher 25(OH)D_3_ than women. When women using oral contraceptives were excluded from the analysis, the association between sex and 25(OH)D_2_ concentration was attenuated (β=0.06; 95% CI −0.002 to 0.13). Conversely, the sex difference still persisted for 25(OH)D_3_ concentrations (β=−0.21; 95% CI −0.26 to −0.15), that is, women having lower concentrations. Low sunlight exposure period (vs high) at sampling associated with higher concentrations of 25(OH)D_2_ but lower concentrations of 25(OH)D_3_. Alcohol abstainers were associated with lower 25(OH)D_3_ concentrations than any other level of drinker. In addition, unhealthy diet score and leisure time computer use were associated with lower 25(OH)D_3_ concentrations.

In sex-stratified analyses, the associations were in the same direction and of similar magnitude with 25(OH)D_2_ and 25(OH)D_3_ concentrations. Female OCP users (vs non-users) had greater serum 25(OH)D_2_ and 25(OH)D_3_ concentrations of 0.17 nmol/L and 0.48 nmol/L, respectively.

Total 25(OH)D associations with potential determinants reflect similar associations as reported for 25(OH)D_3_ concentrations, with the exception of waist circumference and leisure time computer use (table 3). OCP users (vs non-users) were associated with a 0.50 nmol/L greater serum 25(OH)D concentration.

## Discussion

According to the present data collected in 1997, 28% of young adults in Northern Finland were exposed to the risk of vitamin D insufficiency defined by IOM. The average vitamin D status observed in our study was higher than those reported by other studies from the same geographical location (ie, Finland,^27^ ^28^), despite these latter samples being collected after 2002, that is, year of the first Finnish fortification campaign for vitamin D. The mean concentration of serum 25(OH)D measured in both precited studies of the same geographical location (mean age: approx. 37 and 60 years) were nearly 10 nmol/L lower when compared with our population. Our present sample can be considered as a good representation of the young adult population living in Finland at the time of measurement.^29^ In comparison with previous findings, our data may also raise queries about the efficacy of the first wave of fortification introduced in Finland in the year 2002.^8^ The fortification levels were since increased in 2010.^8^ Careful consideration should be made before speculating a potential causation. We must acknowledge, for instance, the differences in study design such as analysis of wider age groups and determination of vitamin D status by radioimmunoassay as opposed to mass spectrometry.

Adding to previous literature, we observed a strong impact of the duration of sunlight in determining the vitamin D status irrespective of the gender.^27^ ^30^ ^31^ The latitude of residence also plays an important role in determining vitamin D status. During the six long winter months in northern latitudes (>60°N), the few hours of daylight are incapable of increasing vitamin D naturally.^6^ The usage of computers outside working hours and a reduced level of physical activity were negatively associated with vitamin D status, which supports previous reports.^30–33^ It is suspected that the observed association between the characteristics of sedentary behaviour in young adults and a lower vitamin D status is likely to be explained by significant changes in the time spent outdoors.^30^ ^32^ ^33^ Unfortunately, the current study does not distinguish between indoor and outdoor physical activity that would help to ascertain this hypothesis. In addition, our results supported the negative association between vitamin D status and obesity or higher waist circumference.^1^ ^27^ ^32^ ^33^ The current hypotheses linking obesity and reduced vitamin D status consider either an effect due to an increased capacity of storage of vitamin D in the fat tissue or the interplay with autocrine factors produced by the adipose tissues.^2^ ^34^ The experimental evidence from animal and human studies is suggesting a direct biological pathway, although the question of reverse causality has not been fully addressed.^1^ ^2^ Currently, the epidemiological data in adults is supporting a causal inference of increased BMI in the reduction of vitamin D status while the reverse has not been confirmed.^1^ In addition, unhealthy diet was negatively associated with vitamin D status. Unfortunately, the food questionnaire used in the present study could not discriminate precisely the consumption of fatty fish or mushrooms to account for a precise dietary quantity of vitamin D3 and D2, respectively. Diet score has been previously examined in the same sample as an adequate proxy of a healthy or unhealthy diet,^23^ but future research with precise food frequency questionnaire is warranted. This will help understand the role of the natural source of dietary vitamin D to reinforce maintenance of a healthy dietary intake whenever possible.

Many reports and reviews consider vitamin D status as a mere representation of individual lifestyle and health behaviour.^35^ The positive association between vitamin D status and the use of OCP is in contrast with the suggestion that vitamin D status merely bio-marks a healthy status. In fact, OCP was linked to 10% higher vitamin D status as consistently reported.^36^ ^37^ Similarly, one study which examined the effect of hormonal contraceptives during vitamin D supplementation in premenopausal women reported that the use of exogenous oestrogen would enhance the response to supplementation.^38^ It is not apparent what the underlying mechanism is pertaining to a higher vitamin D status in women using OCP. Two hypotheses are currently being examined to understand such association. These examine whether the mechanisms by which oestrogen increases the 25(OH)D are due to higher activity of vitamin D 25-hydroxylase in the liver,^39^ or an increase in circulating concentration of vitamin D binding protein (DBP).^37^ According to the IOM classification, OCP users in our study are more likely to be classified as vitamin D sufficient. Previous research using the same data has shown a link between the use of OCP and inflammation.^26^ It will therefore be essential to analyse the pathways underpinning the role of OCP in simultaneously increasing inflammation and vitamin D status. Based on evidence from this and other studies reporting consistently higher vitamin D status in women using OCP, it may be important to implement a corrective factor to the IOM criteria to avoid overestimation of vitamin D status in this subgroup of women.

### Importance of considering D3 and D2 isoforms

Public health recommendations and clinical diagnostics do not currently distinguish between vitamin D2 and D3.^10^ However, there is disagreement on whether these two forms should be considered equivalent.^10^ ^40^ Additionally, 25(OH)D_3_ accounted for the vast majority (>90%) of the circulating 25(OH)D concentrations in the present population. Our study and the study performed by Tolppanen *et al*^31^ were in agreement on the reported associations between the season of blood sampling and the concentrations in 25(OH)D_2_ and 25(OH)D_3_. The determinants associated with the vitamin D status also influenced the serum concentrations of 25(OH)D_3_, with the highest effect being exerted by the season. Importantly, we replicated the associations of the seasonal variation but not the SEP as first observed in children (mean age 9.8 years) of the Avon Longitudinal Studies of Parents and Children.^31^ As expected, 25(OH)D_3_, known as the main contributor of vitamin D status obtained from sunlight, was positively associated with the season of blood sampling and latitude of residence. Interestingly, we observed a heightened vitamin 25(OH)D_2_ status during the winter months that has yet to be understood. However, we do not have information on supplement use which hinders the ability to assess the increased vitamin 25(OH)D_2_ status during winter. As suggested by Tolppanen and colleagues, if serum vitamin D2 is largely associated with dietary and some socioeconomic related factors, this may provide an indication of compensatory behaviour which can be adopted to correct the vitamin D status during the low sunlight months.^31^

## Conclusions and implications

Our results have provided information on the potential determinants associated with the vitamin D status prior to the implementation of a nationwide fortification policy. Understanding the associations between sex, season, latitude and multiple lifestyle factors with dual sources of vitamin D (25(OH)D_2_ and 25(OH)D_3_) will help better understand the role of vitamin D in research, clinical and public health implications. The data also supported a differential association of 25(OH)D_2_ and 25(OH)D_3_ concentrations with sunlight which might have an impact on future strategy for supplementation. These differential results also question current strategies of vitamin D supplementation and IOM cutoffs for vitamin D sufficiency and warrant a personalised approach, accounting for individual and lifestyle characteristics. The fortification of fluid milk products (0.5 μg/100 g) was introduced in Finland in 2002 with limited efficiency in all age groups.^8^ More recently, in April 2010, the fortification levels have been raised further (1.0 μg/100 g).^8^ In addition, in 2012, the Nordic and Finnish nutritional experts have recommended 10 μg/day for all individuals aged 6 months to 75 years, in addition to dietary intake.^41^ Our intended follow-up study from NFBC1966 at 46 years,^42^ will be helpful in measuring the efficiency of waves of fortification before (1997) and after (2012), taking into account multiple determinants and personal supplement use in Northern Finland.
