# Supplementary figures and images for: Potential determinants of vitamin D in Finnish adults: a cross-sectional study from the Northern Finland birth cohort 1966
Source: BMJ Open. 2017 Mar 6;7(3):e013161. doi: 10.1136/bmjopen-2016-013161 (PMC5353308; doi:10.1136/bmjopen-2016-013161)

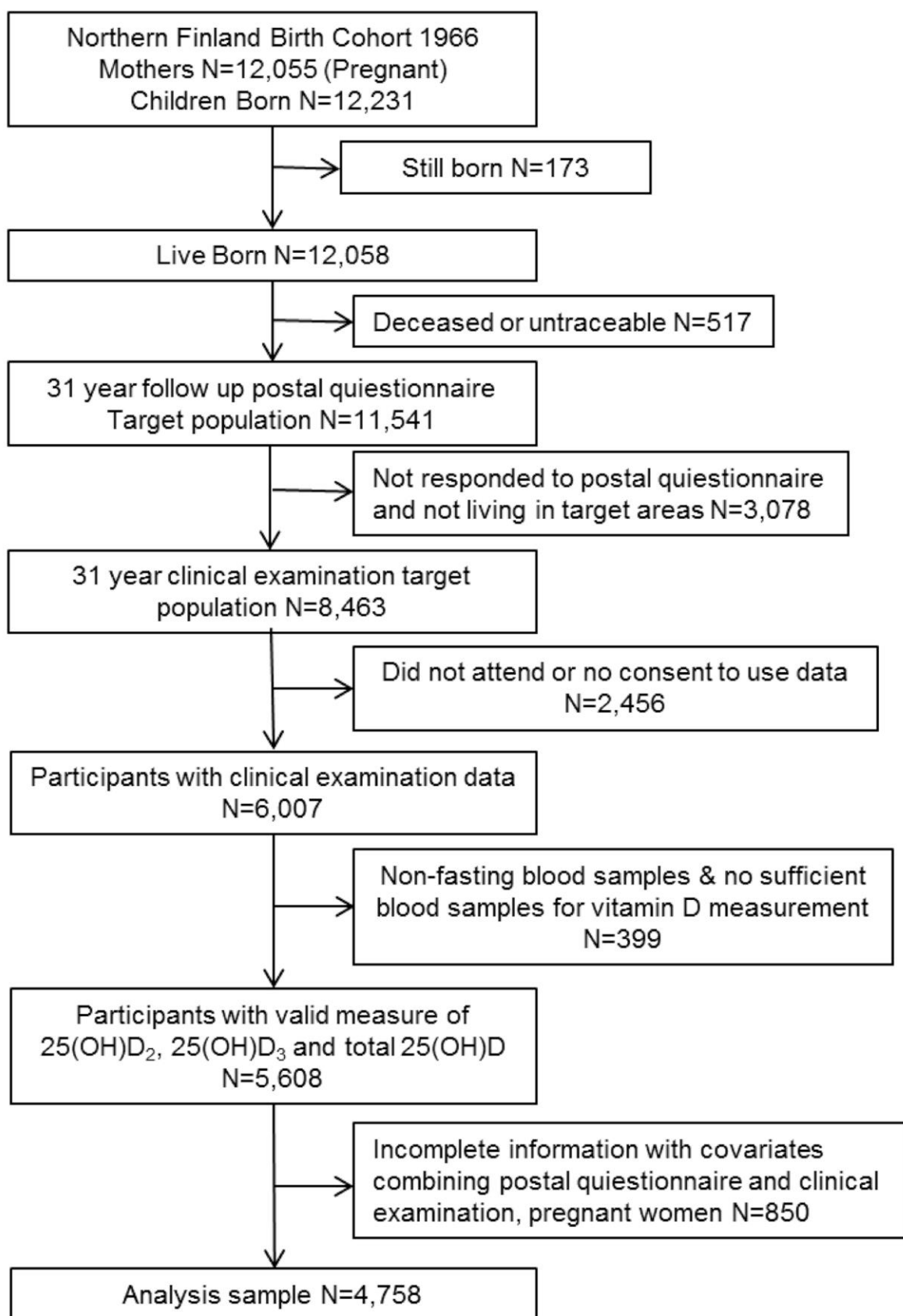

Supplement: supplementary figure [file bmjopen-2016-013161supp_figure.pdf]
