# Supplementary material for: Potential determinants of vitamin D in Finnish adults: a cross-sectional study from the Northern Finland birth cohort 1966
Source: BMJ Open. 2017 Mar 6;7(3):e013161. doi: 10.1136/bmjopen-2016-013161 (PMC5353308; doi:10.1136/bmjopen-2016-013161)
Supplement: supplementary tables [file bmjopen-2016-013161supp_tables.pdf]

Supplementary table 1: The characteristic of male NFBC 1966 participants (N=2,374) in the present study at 31y by serum 25-hydroxyvitamin D tertiles* (I=the lowest tertile; III=the highest tertile).

Tertile of serum 25(OH)D†	I		II		III		
N	782		800		792		
	n or Mean	% or 95 % CI	n or Mean	% or 95 % CI	n or Mean	% or 95 % CI	*P*
Season of blood drawn‡ n %							
High sunlight	292	19.5	514	34.2	695	46.3	<.001
Low sunlight	490	56.1	286	32.8	97	11.1	
Latitude§ n %							
65°N	104	22.6	149	32.4	207	45.0	<.001
>65°N	468	29.8	540	34.4	563	35.8	
Anthropometry:							
Body Mass index (kg/m^2) Mean 95% CI	25.2	24.9- 25.5	25.2	24.9- 25.4	25.2	24.9- 25.4	0.932
Waist circumference (cm) Mean 95% CI	89.6	88.9- 90.3	88.7	88.1- 89.4	88.4	87.7- 89.1	0.055
Socioeconomic position n %							
I + II (Professional)	235	35.9	220	33.7	198	30.4	0.172
III (Skilled worker)	144	33.3	139	32.1	150	34.6	
IV (Unskilled worker)	273	31.9	304	35.5	279	32.6	
V (Farmer)	38	34.2	35	31.5	38	34.3	
VI (Other)	92	28.7	102	31.7	127	39.6	
Lifestyle factors:							
Smoking n %							
Non-smoker	327	34.4	313	32.9	312	32.7	0.301
Former/occasional smoker	180	30.0	202	33.7	218	36.3	
Active smoker	275	33.5	285	34.7	262	31.8	
Alcohol consumption (g/day) n %							
Abstainer	69	36.1	69	36.1	53	27.8	0.539
Low risk drinker	661	32.7	677	33.4	688	33.9	
At-risk drinker	52	33.1	54	34.4	51	32.5	
Leisure time computer use n %							
Never	253	29.7	312	36.6	287	33.7	0.004
No more than once per week	89	28.5	103	33.0	120	38.5	
On 2 to 5 days per week	237	36.2	196	29.9	223	33.9	
On more than 5 days per week	203	36.6	189	34.1	162	29.3	
Quartile of physical activity (MET hours per week) n %							
QI: 0.0 - 3.79	255	37.4	231	33.9	196	28.7	0.001
QII: 3.80 - 11.29	174	30.9	204	36.4	184	32.7	
QIII: 11.30 - 21.99	199	35.2	174	30.7	193	34.1	
QIV: >22.0	154	27.3	191	33.9	219	38.8	
Diet score n %							
0-1	157	34.7	135	29.8	161	35.5	0.150
2-3	485	31.7	532	34.7	514	33.6	
4-5	140	35.9	133	34.1	117	30.0	

MET, metabolic equivalent of task of physical activity;
The values are expressed as mean and 95 % confidence intervals; numbers and %.

* Differences between males and females were tested with ANOVA for normally distributed variables and Pearson's χ^2 test for categorical variables.

† Mean (95% CI) of 25-Hydroxyvitamin D tertiles for male were 41.76 (41.21, 42.31), 63.70 (63.24, 64.16) and 100.90 (99.15, 102.65). Serum total 25(OH)D may differ slightly from the actual sum of D2 and D3 because of amendment of undetectable D2 values (see methods).

‡ The season of blood sampling were categorised as high sunlight [summer (1 June - 30 August), autumn (1 September - 31 October)] and low sunlight [winter (1 November - 31 March) and spring (1 April - 31 May)].

§ Data included only on samples taken during all seasons from Oulu city and other provinces of Oulu and Lapland. Data not included on N=343 in males and N=419 in females with samples taken during winter months from Helsinki region

Supplementary table 2: The characteristic of female NFBC 1966 participants (N=2,384) in the present study at 31y by serum 25-hydroxyvitamin D tertiles* (I=the lowest tertile; III=the highest tertile).

Tertile of serum 25(OH)D†	I		II		III		
N	810		789		785		
	n or Mean	% or 95 % CI	n or Mean	% or 95 % CI	n or Mean	% or 95 % CI	*P*
Season of blood drawn‡ n %							
High sunlight	274	18.9	498	34.3	680	46.8	<.001
Low sunlight	536	57.5	291	31.2	105	11.3	
Latitude§ n %							
65°N	106	24.6	156	36.2	169	39.2	<.001
>65°N	455	29.7	502	32.7	577	37.6	
Anthropometry:							
Body Mass index (kg/m^2) Mean 95% CI	24.4	24.1- 24.7	24.4	24.0- 24.7	23.7	23.4- 23.9	0.002
Waist circumference (cm) Mean 95% CI	79.7	78.9- 80.6	79.2	78.3- 80.1	77.3	76.6- 78.0	<.001
Socioeconomic position n %							
I + II (Professional)	186	38.7	154	32.0	141	29.3	0.046
III (Skilled worker)	357	34.0	364	34.7	329	31.3	
IV (Unskilled worker)	113	30.3	123	33.1	136	36.6	
V (Farmer)	22	40.7	14	25.9	18	33.4	
VI (Other)	132	30.9	134	31.4	161	37.7	
Lifestyle factors:							
Smoking n %							
Non-smoker	415	35.4	373	31.7	388	32.9	0.133
Former/occasional smoker	186	30.3	236	38.4	192	31.3	
Active smoker	209	35.2	180	30.3	205	34.5	
Alcohol consumption (g/day) n %							
Abstainer	96	40.9	77	32.7	62	26.4	0.020
Low risk drinker	674	33.3	672	33.1	681	33.6	
At-risk drinker	40	32.8	40	32.8	42	34.4	
Leisure time computer use n %							
Never	284	33.2	287	33.5	285	33.3	0.051
No more than once per week	119	31.4	131	34.6	129	34.0	
On 2 to 5 days per week	250	32.8	251	32.9	262	34.3	
On more than 5 days per week	157	40.7	120	31.1	109	28.2	
Quartile of physical activity (MET hours per week) n %							
QI: 0.0 - 3.79	189	35.6	163	30.6	180	33.8	0.013
QII: 3.80 - 11.29	229	36.5	217	34.6	181	28.9	
QIII: 11.30 - 21.99	216	33.9	223	35.0	198	31.1	
QIV: >22.0	176	29.9	186	31.7	226	38.4	
Diet score n %							
0-1	321	31.9	342	33.9	345	34.2	0.284
2-3	427	35.4	388	32.1	393	32.5	
4-5	62	36.9	59	35.2	47	27.9	
Contraception‖ n %							
No contraception	441	38.2	401	34.8	312	27.0	<.001
Other kinds of contraception	216	36.6	187	31.6	188	31.8	
Oral contraceptive pills	140	23.1	190	31.3	277	45.6	

MET, metabolic equivalent of task of physical activity;

The values are expressed as mean and 95 % confidence intervals; numbers and %.

* Differences between males and females were tested with ANOVA for normally distributed variables and Pearson's χ^2 test for categorical variables.

† Mean (95% CI) of 25-Hydroxyvitamin D tertiles for female were 41.24 (40.69, 41.80), 64.04 (63.59, 64.50) and 99.12 (97.47, 100.77). Serum total 25(OH)D may differ slightly from the actual sum of D2 and D3 because of amendment of undetectable D2 values (see methods).

‡ The season of blood sampling were categorised as high sunlight [summer (1 June - 30 August), autumn (1 September - 31 October)] and low sunlight [winter (1 November - 31 March) and spring (1 April - 31 May)].

§ Data included only on samples taken during all seasons from Oulu city and other provinces of Oulu and Lapland. Data not included on N=343 in males and N=419 in females with samples taken during winter months from Helsinki region.

‖ Analysis on N=2,352 individuals due to N=32 observations missing with contraception.

Supplementary table 3. The risk of being at lower tertile of total 25-hydroxyvitamin D by factors of interest. Unadjusted and mutually adjusted associations from multinomial ordinal logistic regression analyses*

Explanatory Variables	Total†				Male‡				Female§			
	OR	95% CI	OR	95% CI	OR	95% CI	OR	95% CI	OR	95% CI	OR	95% CI
	Unadjusted		Adjusted‖		Unadjusted		Adjusted‖		Unadjusted		Adjusted‖	
Sex												
Males	Reference		Reference									
Females	1.03	0.93, 1.15	1.09	0.97, 1.24								
global *P* value		0.53		0.13								
Environmental factors:												
Season of blood sampling ††												
High sunlight	Reference		Reference		Reference		Reference		Reference		Reference	
Low sunlight	5.98	5.31, 6.74	2.09	1.80, 2.43	5.79	4.89, 6.86	2.12	1.72, 2.62	6.18	5.22, 7.32	2.18	1.74, 2.74
global *P* value		<.0001		<.0001		<.0001		<.0001		<.0001		<.0001
Latitude												
65°N	Reference		Reference		Reference		Reference		Reference		Reference	
>65°N	4.03	3.44, 4.72	2.03	1.51, 2.73	4.23	3.34, 5.36	2.25	1.49, 3.42	3.88	3.13, 4.82	2.14	1.37, 3.34
global *P* value		<.0001		<.0001		<.0001		0.0001		<.0001		0.0008
Anthropometry:												
Obesity (yes vs No)	1.38	1.15, 1.66	1.35	1.08, 1.68	1.26	0.97, 1.64	1.33	0.96, 1.85	1.51	1.16, 1.95	1.28	0.94, 1.74
global *P* value		0.0005		0.0078		0.088		0.082		0.0018		0.12
Waist Circumference												
M < 94, F < 80	Reference		Reference		Reference		Reference		Reference		Reference	
M ≥ 94, F ≥ 80	1.30	1.16, 1.45	1.22	1.06, 1.40	1.23	1.04, 1.45	1.07	0.87, 1.31	1.35	1.16, 1.57	1.30	1.08, 1.57
global *P* value		<.0001		0.0042		0.015		0.54		0.0001		0.0048
Socioeconomic position												
I+II (Professional)	Reference		Reference		Reference		Reference		Reference		Reference	
III (Skilled worker)	0.88	0.76, 1.01	0.96	0.82, 1.13	0.85	0.68, 1.07	0.97	0.76, 1.23	0.86	0.70, 1.05	0.98	0.78, 1.22
IV (Unskilled worker)	0.81	0.69, 0.94	0.94	0.79, 1.12	0.87	0.72, 1.05	1.01	0.80, 1.26	0.70	0.55, 0.90	0.90	0.68, 1.21
V(Farmer)	0.90	0.67, 1.21	1.01	0.73, 1.40	0.88	0.61, 1.27	0.97	0.64, 1.46	0.96	0.58, 1.62	1.19	0.67, 2.10
VI(Other)	0.70	0.59, 0.83	0.89	0.73, 1.08	0.69	0.54, 0.88	0.89	0.67, 1.18	0.69	0.55, 0.88	0.88	0.67, 1.16
global *P* value		0.0008		0.81		0.057		0.91		0.013		0.77
Lifestyle factors:												
Smoking												
Non-smoker	Reference		Reference		Reference		Reference		Reference		Reference	
Former/occasional smoker	0.88	0.78, 1.01	0.89	0.77, 1.02	0.84	0.69, 1.01	0.85	0.70, 1.05	0.93	0.78, 1.11	0.86	0.71, 1.05
Active smoker	0.98	0.87, 1.11	1.07	0.93, 1.22	1.00	0.84, 1.19	1.09	0.90, 1.32	0.96	0.80, 1.16	0.96	0.78, 1.19
global *P* value		0.15		0.059		0.12		0.08		0.72		0.32
Alcohol consumption (g/day)												
Abstainer	Reference		Reference		Reference		Reference		Reference		Reference	
Low risk drinker	0.76	0.63, 0.91	0.76	0.62, 0.93	0.81	0.61, 1.06	0.76	0.56, 1.02	0.72	0.56, 0.92	0.90	0.68, 1.19
At-risk drinker	0.76	0.58, 1.01	0.60	0.44, 0.81	0.84	0.57, 1.24	0.68	0.44, 1.04	0.70	0.47, 1.04	0.67	0.43, 1.05
global *P* value		0.011		0.003		0.29		0.14		0.03		0.21
Leisure time computer use												
Never	Reference		Reference		Reference		Reference		Reference		Reference	
No more than once per week	0.92	0.78, 1.08	0.89	0.75, 1.06	0.87	0.69, 1.11	0.91	0.71, 1.18	0.95	0.76, 1.18	0.84	0.66, 1.07

On 2 to 5 days per week	1.05	0.92, 1.20	1.06	0.91, 1.23	1.15	0.95, 1.38	1.24	1.00, 1.55	0.97	0.81, 1.16	0.95	0.77, 1.16
On more than 5 days per week	1.30	1.12, 1.50	1.08	0.91, 1.29	1.29	1.06, 1.57	1.17	0.92, 1.49	1.33	1.07, 1.66	1.03	0.79, 1.33
global *P* value		0.0006		0.22		0.0089		0.089		0.025		0.45
Quartile of physical activity (MET-hours per week)												
QI: 0.0 - 3.79	1.42	1.23, 1.65	1.50	1.28, 1.77	1.58	1.28, 1.94	1.60	1.28, 2.01	1.26	1.02, 1.57	1.46	1.15, 1.86
QII: 3.80 - 11.29	1.35	1.16, 1.56	1.34	1.14, 1.57	1.25	1.01, 1.55	1.24	0.99, 1.57	1.44	1.17, 1.77	1.47	1.17, 1.83
QIII: 11.30 - 21.99	1.31	1.13, 1.52	1.26	1.07, 1.48	1.33	1.07, 1.65	1.26	1.00, 1.58	1.29	1.05, 1.59	1.27	1.01, 1.59
QIV: >22.0	Reference		Reference		Reference		Reference		Reference		Reference	
global *P* value		<.0001		<.0001		0.0003		0.0008		0.006		0.0033
Diet score												
Healthy diet	Reference		Reference		Reference		Reference		Reference		Reference	
Unhealthy diet	1.18	1.00, 1.39	1.20	1.00, 1.44	1.19	0.97, 1.45	1.17	0.94, 1.46	1.21	0.91, 1.61	1.23	0.89, 1.69
global *P* value		0.046		0.046		0.094		0.16		0.20		0.22
Contraception												
Oral contraceptive pill (yes vs no)									0.49	0.41, 0.58	0.37	0.30, 0.44
global *P* value										<.0001		<.0001

MET, metabolic equivalent of task of physical activity;

* The values are odds ratios and 95% confidence intervals from ordinal logistic regression analysis by entering each variables separately in unadjusted analysis and by entering all the variables in adjusted analysis.

† Mean (95% CI) of 25-Hydroxyvitamin D tertiles for total sex were 41.50 (41.11, 41.89), 63.87 (63.55, 64.19) and 100.01 (98.81, 101.22). Serum total 25(OH)D may differ slightly from the actual sum of D2 and D3 because of amendment of undetectable D2 values (see methods).

‡ Mean (95% CI) of 25-Hydroxyvitamin D tertiles for male were 41.76 (41.21, 42.31), 63.70 (63.24, 64.16) and 100.90 (99.15, 102.65).

§ Mean (95% CI) of 25-Hydroxyvitamin D tertiles for female were 41.24 (40.69, 41.80), 64.04 (63.59, 64.50) and 99.12 (97.47, 100.77).

‖ Analysis done on N=3,996 (total), N=2,031 (male) and N=1,933 (female). Blood drawn only in winter on N=343 males & N=419 in females residing in Helsinki were excluded. In addition N=32 individuals missing with contraception status in females.

†† The season of blood sampling were categorised as high sunlight [summer (1 June - 30 August), autumn (1 September - 31 October)] and low sunlight [winter (1 November - 31 March) and spring (1 April - 31 May)].

Supplementary table 4. Major factors associated with serum 25(OH)D_2 (Vitamin D2), 25(OH)D_3 (Vitamin D3) and 25(OH)D (Vitamin D) nmol/L concentrations, assessed by univariable and multiple linear regression analysis in male (N=2,374)*

	Serum 25(OH)D_2, nmol/L†				Serum 25(OH)D_3, nmol/L†				Serum 25(OH)D_3, nmol/L†,‖			
	Univariable		Multivariable‡		Univariable		Multivariable‡		Univariable		Multivariable‡	
**Explanatory variables**	β	95% CI	β	95% CI	β	95% CI	β	95% CI	β	95% CI	β	95% CI
**Daylight:**												
Season of blood sampling §												
(Reference: High sunlight)												
Low sunlight	0.55	0.47, 0.63	0.27	0.17, 0.37	-1.02	-1.09, -0.95	-0.44	-0.53, -0.36	-0.93	-1.01, -0.86	-0.38	-0.47,-0.29
global *P* value		<.0001		<.0001		<.0001		<.0001		<.0001		<.0001
Latitude												
(Reference: 65°N)												
>65°N	-0.14	-0.24, -0.04	-0.15	-0.25, -0.05	-0.16	-0.26, -0.06	-0.19	-0.27, -0.10	-0.19	-0.29, -0.09	-0.22	-0.31, -0.13
global *P* value		0.006		0.003		0.002		<.0001		0.0002		<.0001
**Anthropometry:**												
BMI (kg/m^2)												
(Reference : Normal (18.5-24.99))												
Underweight (<18.5)	0.10	-0.30, 0.50	0.14	-0.24, 0.51	-0.23	-0.63, 0.17	-0.23	-0.55, 0.08	-0.24	-0.65, 0.16	-0.23	-0.57, 0.10
Overweight (25–29.99)	-0.09	-0.18, -0.01	-0.03	-0.13, 0.06	0.06	-0.02, 0.15	0.04	-0.03, 0.12	0.05	-0.04, 0.13	0.05	-0.04, 0.13
Obese (≥30)	-0.10	-0.24, 0.05	0.02	-0.16, 0.19	-0.15	-0.30, -0.01	-0.16	-0.31, -0.01	-0.17	-0.32, -0.03	-0.15	-0.31, 0.01
global *P* value		0.12		0.69		0.021		0.0054		0.020		0.056
Waist circumference (cm)												
(Reference: M<94, F<80)												
M≥94, F≥80	-0.09	-0.18, 0.001	-0.09	-0.21, 0.02	-0.12	-0.21, -0.03	-0.02	-0.12, 0.07	-0.14	-0.23, -0.05	-0.05	-0.15, 0.06
global *P* value		0.053		0.12		0.011		0.65		0.0029		0.38
**Socioeconomic position**:												
(Reference: I+II (Professional))												
III (Skilled worker)	-0.12	-0.24, 0.002	-0.10	-0.21, 0.02	0.12	-0.003, 0.24	0.04	-0.06, 0.14	0.09	-0.03, 0.22	0.02	-0.08, 0.12
IV (Unskilled worker)	-0.08	-0.18, 0.02	-0.06	-0.17, 0.05	0.13	0.03, 0.23	0.03	-0.06, 0.12	0.10	0.002, 0.21	0.01	-0.08, 0.11
V(Farmer)	-0.10	-0.30, 0.10	-0.05	-0.25, 0.15	0.14	-0.06, 0.34	0.03	-0.13, 0.20	0.11	-0.09, 0.31	0.02	-0.16, 0.19
VI(Other)	-0.17	-0.30, -0.04	-0.10	-0.24, 0.03	0.25	0.12, 0.38	0.07	-0.05, 0.18	0.22	0.09, 0.35	0.05	-0.07, 0.17
global *P* value		0.11		0.44		0.0053		0.81		0.027		0.96
**Lifestyle:**												
Smoking												
(Reference: Non-smoker)												
Former/occasional smoker	-0.07	-0.17, 0.03	-0.05	-0.14, 0.05	0.05	-0.05, 0.15	0.007	-0.07, 0.09	0.04	-0.06, 0.15	0.002	-0.08, 0.09
Active smoker	-0.09	-0.19, -0.003	-0.04	-0.13, 0.05	-0.02	-0.12, 0.07	-0.07	-0.15, 0.006	-0.04	-0.14, 0.05	-0.08	-0.16, 0.003
global *P* value		0.11		0.52		0.36		0.12		0.27		0.11
Alcohol consumption (g/day)												
(Reference: Abstainer)												
Low risk drinker	0.09	-0.06, 0.23	0.11	-0.03, 0.25	0.10	-0.05, 0.24	0.09	-0.03, 0.21	0.13	-0.02, 0.28	0.12	-0.001, 0.25
At-risk drinker	0.03	-0.17, 0.24	0.07	-0.13, 0.27	0.04	-0.17, 0.25	0.10	-0.07, 0.27	0.06	-0.15, 0.27	0.13	-0.05, 0.31
global *P* value		0.41		0.29		0.37		0.32		0.18		0.1511
Leisure time computer use												

(Reference : Never)												
No more than once per week	0.03	-0.10, 0.16	0.02	-0.10, 0.14	0.01	-0.12, 0.14	-0.02	-0.12,0.09	0.02	-0.11, 0.15	-0.01	-0.12, 0.10
On 2 to 5 days per week	0.03	-0.07, 0.13	-0.03	-0.14, 0.07	-0.09	-0.19, 0.01	-0.07	-0.16, 0.02	-0.07	-0.17, 0.03	-0.07	-0.16, 0.02
On more than 5 days per week	0.05	-0.06, 0.15	-0.07	-0.18, 0.05	-0.20	-0.31, -0.10	-0.10	-0.20,-0.002	-0.20	-0.30, -0.09	-0.12	-0.22, -0.02
global *P* value		0.82		0.59		0.0009		0.19		0.0015		0.11
Quartile of physical activity (MET-hours per week)												
(Reference: QI: 0.0 - 3.79)												
QII: 3.80 - 11.29	0.12	0.01, 0.23	0.12	0.02, 0.23	0.07	-0.04, 0.18	0.05	-0.04, 0.14	0.10	-0.02, 0.21	0.08	-0.02, 0.17
QIII: 11.30 - 21.99	0.06	-0.05, 0.17	0.04	-0.07, 0.14	0.10	-0.01, 0.21	0.10	0.01, 0.19	0.12	0.007, 0.23	0.12	0.02, 0.21
QIV: >22.0	0.12	0.01, 0.23	0.12	0.01, 0.23	0.20	0.08, 0.31	0.14	0.05, 0.23	0.24	0.13, 0.35	0.18	0.09, 0.28
global *P* value		0.094		0.049		0.0066		0.015		0.0004		0.0020
**Diet score**												
(Reference: Healthy diet)												
Unhealthy diet	-0.08	-0.19, 0.03	-0.06	-0.16, 0.05	-0.08	-0.19, 0.03	-0.05	-0.14, 0.03	-0.10	-0.21, 0.01	-0.07	-0.16, 0.03
global *P* value		0.14		0.29		0.14		0.22		0.071		0.16

MET, metabolic equivalent of task of physical activity; 25(OH)D_2, ergocalciferol.

* The values are standardised regression coefficients (β) and *P*-values from linear regression models by entering each variable separately in univariable analysis and by entering all the variables in multivariable analysis.

† 1 SD increase/decrease in 25(OH)D_2, 25(OH)D_3 and 25(OH)D nmol/L per 1 unit or category change in explanatory variable.

‡ Analysis done on N=2,031 (males). Blood drawn only in winter on N=343 males residing in Helsinki were excluded.

§ The season of blood sampling were categorised as high sunlight [summer (1 June - 30 August), autumn (1 September - 31 October)] and low sunlight [winter (1 November - 31 March) and spring (1 April - 31 May)].

‖ Serum total 25(OH)D may differ slightly from the actual sum of D2 and D3 because of amendment of undetectable D2 values (see methods).

Supplementary table 5. Major factors associated with serum 25(OH)D_2 (Vitamin D2), 25(OH)D_3 (Vitamin D3) and 25(OH)D (Vitamin D) nmol/L concentrations, assessed by univariable and multiple linear regression analysis in female (N=2,384)*

	Serum 25(OH)D_2, nmol/L†				Serum 25(OH)D_3, nmol/L†				Serum 25(OH)D, nmol/L†,‖			
	Univariable		Multivariable‡		Univariable		Multivariable‡		Univariable		Multivariable‡	
**Explanatory variables**	β	95% CI	β	95% CI	β	95% CI	β	95% CI	β	95% CI	β	95% CI
**Daylight:**												
Season of blood sampling §												
(Reference: High sunlight)												
Low sunlight	0.59	0.51, 0.67	0.31	0.20, 0.43	-1.03	-1.10, -0.96	-0.43	-0.52, -0.34	-0.90	-0.98, -0.83	-0.34	-0.44, -0.25
global *P* value		<.0001		<.0001		<.0001		<.0001		<.0001		<.0001
Latitude												
(Reference: 65°N)												
>65°N	-0.03	-0.14, 0.08	0.03	-0.08, 0.13	-0.12	-0.23,-0.02	-0.20	-0.29, -0.11	-0.12	-0.23, -0.01	-0.19	-0.28, -0.10
global *P* value		0.59		0.57		0.025		<.0001		0.027		<.0001
**Anthropometry:**												
BMI (kg/m^2)												
(Reference : Normal (18.5-24.99))												
Underweight (<18.5)	-0.12	-0.36, 0.11	-0.10	-0.32, 0.13	-0.02	-0.25, 0.21	0.02	-0.16, 0.20	-0.05	-0.28, 0.18	0.002	-0.18, 0.19
Overweight (25–29.99)	-0.07	-0.17, 0.03	0.03	-0.09, 0.15	-0.06	-0.16, 0.04	-0.07	-0.16, 0.03	-0.09	-0.19, 0.01	-0.08	-0.18, 0.02
Obese (≥30)	-0.16	-0.30, -0.01	-0.02	-0.19, 0.14	-0.23	-0.38, -0.09	-0.12	-0.26, 0.01	-0.27	-0.42, -0.13	-0.14	-0.28, 0.01
global *P* value		0.10		0.72		0.014		0.29		0.0015		0.22
Waist circumference (cm)												
(Reference: M<94, F<80)												
M≥94, F≥80	-0.12	-0.20, -0.03	-0.12	-0.23, -0.01	-0.13	-0.21, -0.05	-0.04	-0.13, 0.05	-0.16	-0.25, -0.08	-0.06	-0.16, 0.03
global *P* value		0.007		0.033		0.002		0.36		0.0002		0.1943
**Socioeconomic position:**												
(Reference: I+II (Professional))												
III (Skilled worker)	-0.05	-0.17, 0.06	-0.02	-0.12, 0.09	0.08	-0.03, 0.19	-0.002	-0.09, 0.08	0.08	-0.03, 0.19	0.01	-0.08, 0.10
IV (Unskilled worker)	-0.004	-0.14, 0.14	0.10	-0.04, 0.24	0.15	0.01, 0.28	-0.02	-0.14, 0.09	0.16	0.02, 0.29	0.02	-0.10, 0.14
V(Farmer)	-0.10	-0.39, 0.19	0.02	-0.26, 0.30	-0.11	-0.40, 0.17	-0.20	-0.43, 0.03	-0.13	-0.42, 0.15	-0.18	-0.42, 0.06
VI(Other)	-0.14	-0.27, -0.004	-0.03	-0.16, 0.11	0.18	0.05, 0.31	0.03	-0.07, 0.14	0.17	0.04, 0.30	0.05	-0.07, 0.16
global *P* value		0.28		0.36		0.026		0.36		0.029		0.44
**Lifestyle:**												
Smoking												
(Reference: Non-smoker)												
Former/occasional smoker	0.02	-0.08, 0.12	0.02	-0.07, 0.12	0.03	-0.06, 0.14	0.05	-0.02, 0.13	0.03	-0.07, 0.13	0.04	-0.04, 0.13
Active smoker	-0.09	-0.19, 0.01	-0.06	-0.16, 0.05	0.03	-0.07, 0.13	0.002	-0.08, 0.08	0.01	-0.09, 0.11	-0.02	-0.10, 0.07
global *P* value		0.12		0.35		0.73		0.35		0.84		0.42
Alcohol consumption (g/day)												
(Reference: Abstainer)												
Low risk drinker	0.01	-0.13, 0.15	0.003	-0.13, 0.14	0.22	0.08, 0.36	0.07	-0.04, 0.18	0.23	0.09, 0.36	0.07	-0.04, 0.19
At-risk drinker	0.07	-0.15, 0.30	0.03	-0.19, 0.25	0.19	-0.03, 0.41	0.16	-0.01, 0.34	0.20	-0.02, 0.42	0.16	-0.03, 0.34
global *P* value		0.81		0.96		0.0066		0.19		0.0044		0.24

Leisure time computer use												
(Reference : Never)												
No more than once per week	0.02	-0.10, 0.15	-0.0004	-0.12, 0.12	0.01	-0.11, 0.13	0.05	-0.04, 0.15	0.01	-0.11, 0.13	0.05	-0.05, 0.15
On 2 to 5 days per week	0.02	-0.08, 0.12	0.02	-0.09, 0.12	0.001	-0.10, 0.10	-0.01	-0.09, 0.07	0.005	-0.09, 0.10	-0.004	-0.09, 0.08
On more than 5 days per week	0.18	0.05, 0.30	0.12	-0.01, 0.24	-0.23	-0.35, -0.11	-0.08	-0.18, 0.02	-0.16	-0.28, -0.04	-0.02	-0.13, 0.08
global *P* value		0.034		0.28		0.0008		0.15		0.032		0.66
Quartile of physical activity (MET-hours per week)												
(Reference: QI: 0.0 - 3.79)												
QII: 3.80 - 11.29	0.03	-0.09, 0.15	-0.04	-0.16, 0.07	-0.11	-0.23, 0.005	-0.03	-0.12, 0.06	-0.12	-0.23, -0.002	-0.05	-0.15, 0.05
QIII: 11.30 - 21.99	0.13	0.01, 0.25	0.05	-0.06, 0.17	-0.06	-0.18, 0.05	0.02	-0.07, 0.11	-0.04	-0.15, 0.08	0.02	-0.07, 0.12
QIV: >22.0	0.09	-0.03, 0.21	0.02	-0.10, 0.14	0.10	-0.02, 0.22	0.13	0.04, 0.23	0.11	-0.008, 0.23	0.12	0.03, 0.22
global *P* value		0.13		0.36		0.0017		0.0027		0.0011		0.0028
Diet score												
(Reference: Healthy diet)												
Unhealthy diet	-0.13	-0.30, 0.03	-0.09	-0.24, 0.07	-0.08	-0.24, 0.08	-0.09	-0.21, 0.04	-0.12	-0.28, 0.04	-0.12	-0.25, 0.01
global *P* value		0.10		0.24		0.31		0.18		0.14		0.081
**Contraception status**												
(Reference: No contraception)												
Other kinds of contraception	0.01	-0.09, 0.11	0.03	-0.07, 0.13	0.10	0.01, 0.20	0.06	-0.02, 0.14	0.11	0.01, 0.21	0.07	-0.02, 0.15
Oral contraceptive pills	0.21	0.11, 0.31	0.17	0.08, 0.27	0.47	0.37, 0.57	0.48	0.41, 0.56	0.50	0.40, 0.60	0.50	0.42, 0.58
global *P* value		<.0001		0.0018		<.0001		<.0001		<.0001		<.0001

MET, metabolic equivalent of task of physical activity; 25(OH)D_2, ergocalciferol.

* The values are standardised regression coefficients (β) and *P*-values from linear regression models by entering each variable separately in univariable analysis and by entering all the variables in multivariable analysis.

† 1 SD increase/decrease in 25(OH)D_2, 25(OH)D_3 and 25(OH)D nmol/L per 1 unit or category change in explanatory variable.

‡ Analysis done on N=1,933 (females). Blood drawn only in winter on N=419 in females residing in Helsinki were excluded. In addition N=32 individuals missing with contraception status in females.

§ The season of blood sampling were categorised as high sunlight [summer (1 June - 30 August), autumn (1 September - 31 October)] and low sunlight [winter (1 November - 31 March) and spring (1 April - 31 May)].

‖ Serum total 25(OH)D may differ slightly from the actual sum of D2 and D3 because of amendment of undetectable D2 values (see methods).
